# Association between Circle of Willis Configuration and Rupture of Cerebral Aneurysms

**DOI:** 10.3390/medicina55070338

**Published:** 2019-07-03

**Authors:** Nebojša N. Stojanović, Aleksandar Kostić, Radisav Mitić, Luka Berilažić, Miša Radisavljević

**Affiliations:** 1 Clinic of Neurosurgery, Clinical Center Niš, Zorana Đinđića 48, 18000 Niš, Serbia; 2 Faculty of Medicine, University of Niš, Zorana Đinđića 81,18000 Niš, Serbia; 3 Institute of Pathology, Clinical Center Niš, Zorana Đinđića 48, 18000 Niš, Serbia

**Keywords:** cerebral aneurysms, circle of Willis

## Abstract

*Background and Objectives:* Intracranial hemorrhage caused by the rupture of brain aneurysms occurs in almost 10 per 100,000 people whereas the incidence of such aneurysms is significantly higher, accounting for 4–9%. Linking certain factors to cerebral aneurysm rupture could help in explaining the significantly lower incidence of their rupture compared to their presence. The aim of this study is to determine the association between the corresponding circle of Willis configurations and rupture of cerebral aneurysms. *Materials and Methods:* A group of 114 patients treated operatively for aruptured cerebral aneurysm and a group of 56 autopsied subjects were involved in the study. Four basic types of the circle of Willis configurations were formed—two symmetric types A and C, and two asymmetric types B and D. *Results:* A statistically significantly higher presence of asymmetry of the circle of Willis was determined in the group of surgically-treated subjects (*p* = 0.001),witha significant presence of asymmetric Type B in this group (*p* < 0.001). The changeson the A1 segment in the group of surgically-treated subjects showed a statistically significant presence compared to the group of autopsied subjects (*p* = 0.001). Analyzing the presence of symmetry of the circle of Willis between the two groups, that is, the total presence of symmetric types A and C, indicated their statistically significant presence in the group of autopsied patients (*p* < 0.001)*. Conclusions:* Changes such as hypoplasia or aplasia of A1 and the resulting asymmetry of the circle of Willis directly affect the possibility of the rupture of cerebral aneurysms. Detection of the corresponding types of the circle of Willis after diagnostic examination can be the basis for the development of a protocol for monitoring such patients.

## 1. Introduction

Intracranial aneurysms are on average asymptomatic until the moment of rupture. Intracranial hemorrhage caused by aneurysm rupture occurs in almost 10 per 100,000 people in the general population [1,2]. However, the presence of cerebral aneurysms is much more common and ranges between 3.6–9% depending on the series [3,4,5,6,7]. From these data, it is clear that there is a big difference between the incidence of cerebral aneurysms and the frequency of their rupture. All this leads us to think about the causes of cerebral aneurysm rupture.

Locations of cerebral aneurysms are typically the sites of arterial junctions on the blood vessels of the base of the brain. The interconnected blood vessels at the base of the brain form a specific ring-like configuration, named after Willis who first described them. There are various forms of interconnected blood vessels within the circle of Willis itself [8]. The perfusion characteristics of each configuration of the circle of Willis directly depend on its anatomical characteristics i.e., the presence of normoplastic, hypoplastic or aplastic parts [9,10]. The presence of such anatomical features causes perfusion load of certain parts of the circle of Willis [11,12].Basically, all configurations of the circle of Willis can be divided into symmetric and asymmetric. By analyzing such blood vessel configurations at the base of the brain in the population with cerebral aneurysms, we can contribute to understanding the complex process of rupture of cerebral aneurysm.

This studyaims at linking the basic circle of Willis configurations with the cerebral aneurysm rupture.

## 2. Material and Methods

The presence of morphological variations in the blood vessels of the base of the brain was monitoredin two groups of subjects. The first group was made up of 114 patients who underwent surgical treatment for ruptured aneurysmal changes in the cerebral blood vessels. The second group was formed from 56 subjects who were subjected to autopsy after a fatal outcome that was not caused by hemorrhagic intracranial disease. The investigation was conducted in the perod from 2012 to 2014 at the Department of Neurosurgery and the Institute of Pathology.

The first group of patients was subjected to MSCT and angiographic imaging of the cerebral blood vessels. Preoperative analysis of angiographic images was made and the existence of corresponding configurations of the circle of Willis was established. A dominant flow was determined in relation to the position of an aneurysm. The corresponding blood vessel diameters were compared to the symmetric blood vessel diameters on the opposite side. Reduction of the blood vessel diameter by 1/3 to 2/3 in relation to the diameter of the blood vessel on the opposite side was marked as hypoplasia, and a decrease in the blood vessel diameter below 1/3 of the thickness of the opposite vessel was marked as pronounced hypoplasia. The presence of hypoplasia determined the symmetry or asymmetry of the circle of Willis. Intraoperatively, the relationship between visualized parts of the circle of Willis and aneurysm was analyzed and then compared with the angiographic finding.

**Figure 1:**
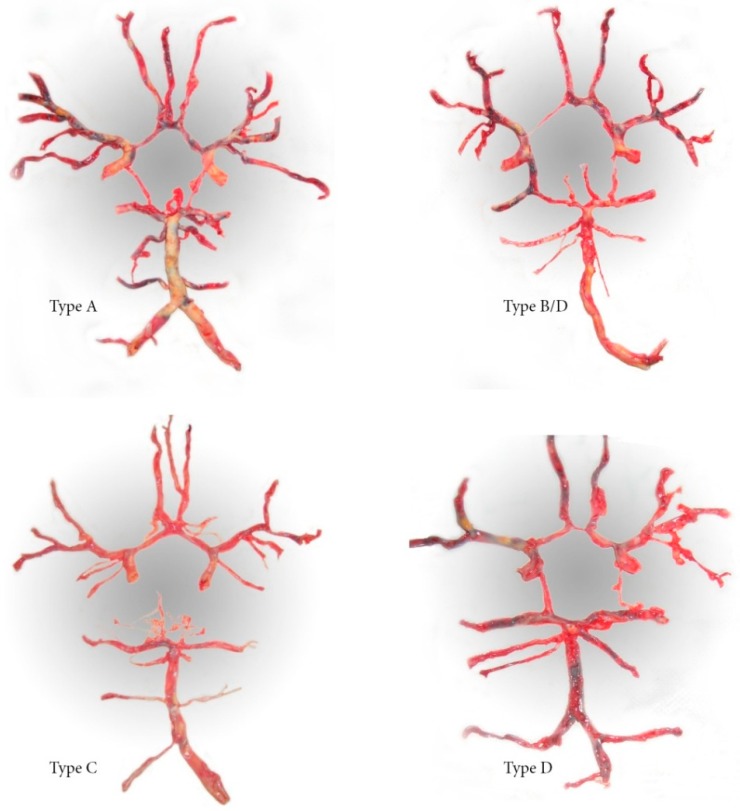
Preparations of the circle of Willis.

The second group consisted of subjects who underwent clinical or postmortem autopsy and who did not die due to some intracranial hemorrhagic disease. The subjects were selected by random sample method. During the autopsy, the brain was taken out from the cranial fossa together with the blood vessels of the base of the skull using a precise technique, and those at the entrance to the cranial cavity were resected. By precise preparation, all blood vessels were separated from the base of the brain and distributed on a homogeneous flat surface with the formation of the typical configuration of the circle of Willis. The thickness of the blood vessels, the presence of anomalies, their arrangement, and the symmetry of the present changes were observed. After suchpreparation, the circles of Willis were photographed by a digital camera and then analyzed on a computer for a possible update of the original finding (Figure 1).

For the purpose of comparing and analyzing the presence of the corresponding configurations of the circle of Willis, four basic types of configuration and one sub-type were formed.

Type A represents a symmetric circle of Willis with different variations at the level of the anterior communicating artery (ACoA).

Type B is an asymmetric circle of Willis with hypoplasia/aplasia of the A1 segment of the anterior cerebral artery (ACA).

Type C represents a symmetric circle of Willis with varying degrees of the hypoplasia/aplasia of the posterior communicating artery (PCoA) bilaterally, or the presence of a bilateral fetal PCoA type.

Type D is an asymmetric circle of Willis with single-sided PCoA hypoplasia or a single-sided fetal PCoA type.

**Figure 2:**
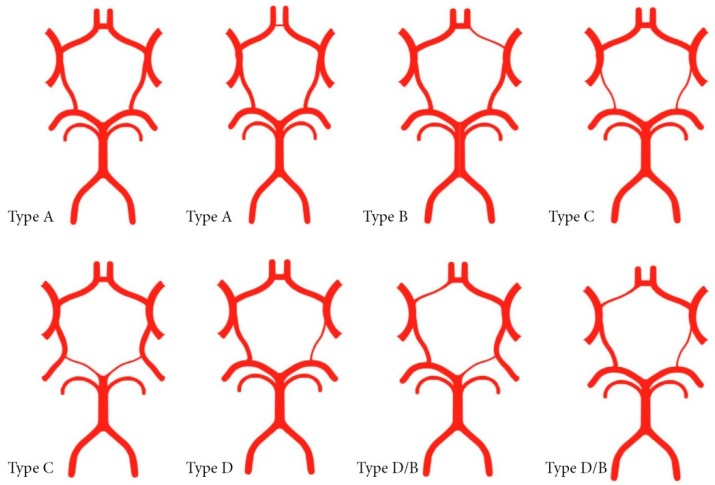
Basic types of configurationof the circle of Willis.

Subtype B/D represents an asymmetric circle of Willis with hypoplasia/aplasia of the A1 segment of the anterior cerebral artery, in combination with changes in the posterior segment (due to hypoplasia of PCoA or PCA) (Figure 2).

The study complies with the Declaration of Helsinki. The study protocol (No. CLI-1000) form was approved by the Ethics Committee of the Clinical Center Niš, Niš, Serbia (3 January 2016). The autopsies were done in accordance with the health care law and the bylaws of the Ministry of Health. The patients undergoing surgery gave verbal consent that the results of their angiographic recording and surgery findings can be used in the study.

### Statistical Analysis

All the findings were numerically processed, tabulated and subjected to statistical analysis of the existing differences between the groups. The chi-squared test was applied for categorical variables, and the exact Fisher test was used when expected frequencies were less than 5. A *p*-value of <0.05 was selected as statistically significant. Statistical analysis was performed in EPI INFO v7.2.2.6 (CDC, Atlanta, GA, USA).

## 3. Results

In the group of autopsied patients, there was a total of 15 (26.8%) asymmetries of the circle of Willis, while 73 (64%) asymmetric configurations were found in the group of surgically-treated patients. After comparing the presence of asymmetry of the circle of Willis (Type B and Type D) among the groups, it was found to be statistically significant in the group of surgically-treated subjects (*p* < 0.001) (Table 1).

By comparing the relationship between the representation of changes in the posterior segment and PCoA and its impact on the occurrence of asymmetry of the circle of Willis, no statistical difference was found between thegroup of subjects undergoing surgery and the group of autopsied subjects (*p* = 0.737) (Table 1).

The presence of symmetric Type A and type B was statistically different between the three groups (*p* = 0.001, *p* < 0.001, respectively) (Table 2).

The symmetric Type A was significantly more frequent in the autopsied patients as compared to patients with a solitary aneurysm (*p* = 0.008) and multiple aneurysms (*p* = 0.004). The asymmetric Type B was significantly less frequent in the autopsied patients as compared to patients with a solitary aneurysm (*p* = 0.002) and multiple aneurysms (*p* < 0.001). The presence of symmetric Type C and asymmetricTypeD did not show any statistical differences between the groups.

In the group of patients with multiple aneurysms, the presence of asymmetric Type B was more frequent than in the group with solitary aneurysms, but without statistical significance (*p* = 0.471, Table 3).

The presence of asymmetric type B in the group of patients operated on was observed in 50 (43.9%) patients. Of these, 31 (62%) patients were with right-sided hypoplasia of the A1 segment, and 19 (38%) with left-sided A1 hypoplasia. Thirty-six (72%) ruptured aneurysms which were associated with hypoplasia of the A1 segment were localized on the ACoA. If we consider only the associationbetween A1 hypoplasia and rupture of the aneurysm on AcoA, it can be seen that 70% of cases show the presence of right-sided hypoplasia.Fourteen (28%) ruptured aneurysms associated with A1 hypoplasia were at other locations (MCA, ICA, PCoA, perA), with 12 of them being positioned on the side of the hypoplasticsegment.However, no statistical significance was found between the hypoplasia of the A1 segment and the segment with a ruptured aneurysm (Table 4).

In the group of 114 surgically-treated patients, the presence of right-sided A1 hypoplasia was recorded in 27.2% (31 subjects) of patients, and of left-sided hypoplasiain 16.7% (19 subjects), while in the group of 56 autopsied subjects,7.1% (4 subjects)were with left-sided hypoplasia of the A1 segment and 8.9% (5 subjects) with right-sided hypoplasia. Overall, A1 segment hypoplasia was significantly more frequent among surgically-treated patients compared to autopsied patients (43.8% vs. 16.1%, *p* < 0.001).

## 4. Discussion

Our results suggest that asymmetric configuration of the circle of Willis was much more frequent in the patients operated on after the rupture of an aneurysm than in subjects from the autopsy. It has also been established that there is a statistically significant presence of hypoplasticchanges on the A1 segment in the group of patients undergoing surgery, which correlates with other studies [13,14,15]. The obtained results indicateda strong link between Type B configuration and aneurysm rupture, as well asits high hemodynamic instability.

This finding indicates that the morphological characteristics of the type of hypoplasia or aplasia in the anterior segment lead to an increase in blood flow from the opposite side and a compensatory increase in flow through ACoA [9,16]. Increasing intramural pressure on the connective parts of the ACoA complex can initiate the process of remodeling the blood vessel resulting in the formation of an aneurysmal sacand then to its rupture [17].

Unlike the anterior segment, changes in the posterior segment of the circle of Willis have not shown significant association with the formation of cerebral aneurysms. During embryogenesis and with the development and differentiation of the carotid and basilar basin, the posterior communicating artery has a tendency forregression and decrease of blood flow [18]. Its functioning at the zero-flowlevel [19] indicates that stress at its exit from the carotid artery is not caused by blood redistribution, but by other hemodynamic disorders. This is also suggested by the fact that despite identical characteristics of the PCoA junction with ICA and PCA, aneurysms occur primarily on the carotid portion of PCoA. The dependence of the formation of aneurysms on PCoA from hemodynamic relationships in the ICA itself is clearly noticed in cases of the fetal type ofPCoA, the existence of which has not shown significant association with the formation of aneurysms. If we observe the point of separation of PCoA in relation to ICA, as a point of bifurcation angle, then we can apply the principle of the optimal distribution of flow for the given angle [10]. Any increase in this angle leads to a direct increase in stress on the site of separation of PcoA, so that the formation of an aneurysm on PcoA is due to the geometry itself and the course the ICA takes in thatpart of PcoA [20,21].

The significant prevalence of symmetric Type A in the group of autopsied subjects (in correlation with other studies) [22,23] and the statistically significant presence of asymmetric Type B in the group of subjects operated on due to cerebral aneurysm rupture clearly points tothe association between the configuration of the circle of Willis with the formation and rupture of cerebral aneurysms.

The presence of C and D configurations in the tested groups did not show a significant link with the formation and rupture of cerebral aneurysms. All this indicates that the presence or absence of PCoA changes cannot be associated with the onset or rupture of cerebral aneurysms.

The significant presence of the right-sided hypoplastic A1 in the group of patients undergoing surgery is interesting. The tendency of types B and D to right-sided positioning of the morphological variants of A1 segment can be considered as part of the embryologic development of the vascular network and perfusion needs of the corresponding brain hemispheres.

In an experimental study with rabbits, Ersin et al. demonstrated the hemodynamic correlation betweenincreased perfusion requirements on the formation of aneurysms [23], and Guangyu Zhu et al. pointed to the significance of collateral circulation within different anatomical variations of the circle of Willis [16]. Ren et al. mathematically provedthe association between anatomical variationsof the circle of Willis on its perfusion characteristics [24]. Just like in our study, in a study conducted by deRooijet al., the configuration of the circle of Willis was identified as a risk factor for the cerebral aneurysm rupture, suggesting that ruptures are also associated withthe direction of blood flow and the shape of aneurysms [25].

The haemodynamic load of the asymmetric configurations of the circle of Willis, which occurs during the achievement of adequate perfusion, can be periodic or permanent. Continuous load, however, can lead to dilatation and occurrence of aneurysmal changes in certain parts of the brain’s circulation system.

## 5. Conclusions

Asymmetric configuration basically has increased perfusion requirements in certain parts, which in turn can initiate the process of blood vessel remodeling in the part of permanently increased perfusion flow. Remodeling can be directed towards achieving an appropriate expansion of collateral circulation and adequate perfusion while reducing the existing stress in the loaded parts. Any further increase in perfusion requirements can lead to the continuation of the vessel wall remodeling cascade and dilatation at the site of the greatest stress, and to the gradual formation of an aneurysmal sac.

Hypoplasia of the A1 segment implies significantly disturbed perfusion relationships in the AcomA area and the stress burden of the junction angle between A1 and A2, which in turn prompts the formation of an aneurysmal sac.

The asymmetric circle of Willis configurations significantly increases the risk of brain aneurysm rupture.

Formation of the basic circle of Willis configuration types and association with their susceptibility to cerebral aneurysm rupture can be used to make a protocol for monitoring unruptured aneurysms that are detected after MSCT or NMR angiography.

## Figures and Tables

**Table 1:** Configuration of the circle of Willis.

Configuration of the Circle of Willis		Autopsied Patients *n* = 56	Surgically Treated Patients *n* = 114	*p*-Values ^1^
Symmetric Willis—Type A and Type C		41(73.2%)	41 (36.0%)	<0.001
Asymmetric Willis—Type B and Type D		15 (26.8%)	73 (64.0%)	
Asymmetric Willis—Type B	Changes in the A1 segment	9(16.0%)	50 (43.9 %)	0.737
Asymmetric Willis—Type D	Changes in the posterior segments	6(10.8%)	23 (20.2%)	

^1^ Chi-squared test.

**Table 2:** Types of configuration of the circle of Willis—the group of autopsied subjects and surgically-treated subjects.

Type Circle of Willis	Autopsied Patients		Surgically Treated Patients				*p*-Value ^1^
			Solitary Aneurysm		Multiple Aneurysms		
Type A	27	(48.2%)	20	(24.7%) ^2^	5	(15.2%) ^2^	0.001
Type B	4	(16.0%)	32	(42.0%) ^2^	15	(51.5%) ^2^	<0.001
subtype B/D	5		2		2		
Type C	14	(25.0%)	13	(16.0%)	3	(9%)	0.143
Type D	6	(10.8%)	14	(17.3%)	8	(24.3%)	0.242
Σ	56	(100%)	81	(100.0%)	33	(100%)	

^1^ Chi squared test, ^2^ *p* < 0.05 vs. autopsied patients.

**Table 3:** Types of circle of Willis configuration—agroup of patients with multiple aneurysms and solitary aneurysms.

**Types Willis**	**Multiple Aneurysms** **Locations of Ruptured Aneurysms**						**Σ**
	**ACoA**	**MCA**	**ICA**	**PCoA**	**perA**	**VBA**	
Type A	0	3	1	1	0	0	5 (15.2%)
Type B	12	0	3	0	0	0	17 (51.5%)
Sub type B/D	0	0	0	2	0	0	
Type C	0	1	2	0	0	0	3 (9.0%)
Type D	0	6	0	2	0	0	8 (24.3%)
Σ	12	10	6	5	0	0	33 (100.0%)
**Type Willis**	**Locations of Solitary Ruptured Aneurysms**						**Σ**
	**ACoA**	**MCA**	**ICA**	**PCoA**	**perA**	**VBA**	
Type A	9	5	2	2	1	1	20 (24.7%)
Type B	24	3	2	0	3	0	34 (42.0%)
subtype B/D	0	0	0	2	0	0	
Type C	0	8	2	2	0	1	13 (16.0%)
Type D	0	4	24	6	0	0	14 (17.3%)
Σ	33	20	10	12	4	2	81 (100.0%)

ACoA, anterior communicative artery; MCA, medial cerebral artery; ICA, internal carotid artery; PCoA, posterior communicative artery; perA, pericalosis artery; VB, vertebrobasilar artery.

**Table 4:** Relationshipbetweenhypoplasia of A1and location of the ruptured aneurysm.

Locations Ruptured Aneurysms	Ruptured Aneurysm in the Surgically Treated Group	Hypoplasia A1 Right	Hypoplasia A1 Left	*p*-Value ^1^
ACoA	36 (72%)	25 (80.6%)	11 (57.9%)	0.157
MCA	3 (6%)	1 (3.2%)	2 (10.5%)	0.549 ^2^
ICA	5 (10%)	1 (3.2%)	4 (21.1%)	0.062 ^2^
PCoA	3 (6%)	2 (6.5%)	1 (5.3%)	1.000 ^2^
perA	3 (6%)	2 (6.5%)	1 (5.3%)	1.000 ^2^
Surgically treated group	50 (43.8%) ^3^	31 (62%)	19 (38%)	<0.001 ^1,4^
Autopsied group	9 (16.1%) ^3^	5 (55.6%)	4 (44.4%)	

^1^ Chi-squared test, ^2^ Fisher’s test, ^3^ percentages related to the total number of surgically treated and autopsied patients, ^4^ comparison of hypoplasia A1 between surgically treated and autopsied patients.
